# A second major histocompatibility complex susceptibility locus for multiple sclerosis

**DOI:** 10.1002/ana.21063

**Published:** 2007-03

**Authors:** Tai Wai Yeo, Philip L De Jager, Simon G Gregory, Lisa F Barcellos, Amie Walton, An Goris, Chiara Fenoglio, Maria Ban, Craig J Taylor, Reyna S Goodman, Emily Walsh, Cara S Wolfish, Roger Horton, James Traherne, Stephan Beck, John Trowsdale, Stacy J Caillier, Adrian J Ivinson, Todd Green, Susan Pobywajlo, Eric S Lander, Margaret A Pericak-Vance, Jonathan L Haines, Mark J Daly, Jorge R Oksenberg, Stephen L Hauser, Alastair Compston, David A Hafler, John D Rioux, Stephen Sawcer, The International Multiple Sclerosis Genetics Consortium

**Affiliations:** 1 Department of Clinical Neurosciences, University of Cambridge, Addenbrooke's HospitalCambridge, United Kingdom; 2 Department of Neurology, Center for Neurologic Diseases, Brigham and Women's HospitalBoston; 3 Harvard Medical SchoolBoston; 4 Program in Medical and Population Genetics, The Broad Institute at the Massachusetts Institute of Technology and Harvard UniversityCambridge, MA; 5 Duke University Medical Center, Center for Human GeneticsDurham, NC; 6 Department of Neurology, School of Medicine, University of California San FranciscoSan Francisco; 7 Division of Epidemiology, School of Public Health, University of California at BerkeleyBerkeley, CA; 8 Department of Neurological Sciences, Dino Ferrari Center, University of Milan, IRCCS Ospedale Maggiore PoliclinicoMilan, Italy; 9 Tissue Typing Laboratory, Addenbrooke's HospitalUnited Kingdom; 10 Wellcome Trust Sanger Institute, Genome CampusHinxton, United Kingdom; 11 Department of Pathology, Immunology Division, University of CambridgeCambridge, United Kingdom; 12 Harvard Center for Neurodegeneration and RepairBoston, MA; 13 The Center for Genome Research, Massachusetts General HospitalBoston, MA; 14 Center for Human Genetics Research, Vanderbilt University Medical CenterNashville, TN; 15 Institute for Human Genetics, School of Medicine, University of California San FranciscoSan Francisco, CA; 16 Montréal Heart Institute and Université de MontréalMontréal, Québec, Canada

## Abstract

**Objective:**

Variation in the major histocompatibility complex (MHC) on chromosome 6p21 is known to influence susceptibility to multiple sclerosis with the strongest effect originating from the *HLA*-*DRB1* gene in the class II region. The possibility that other genes in the MHC independently influence susceptibility to multiple sclerosis has been suggested but remains unconfirmed.

**Methods:**

Using a combination of microsatellite, single nucleotide polymorphism, and human leukocyte antigen (HLA) typing, we screened the MHC in trio families looking for evidence of residual association above and beyond that attributable to the established DRB1*1501 risk haplotype. We then refined this analysis by extending the genotyping of classical HLA loci into independent cases and control subjects.

**Results:**

Screening confirmed the presence of residual association and suggested that this was maximal in the region of the *HLA*-*C* gene. Extending analysis of the classical loci confirmed that this residual association is partly due to allelic heterogeneity at the *HLA*-*DRB1* locus, but also reflects an independent effect from the *HLA*-*C* gene. Specifically, the *HLA*-C*05 allele, or a variant in tight linkage disequilibrium with it, appears to exert a protective effect (*p* = 3.3 × 10^−5^).

**Interpretation:**

Variation in the *HLA*-*C* gene influences susceptibility to multiple sclerosis independently of any effect attributable to the nearby *HLA*-*DRB1* gene. Ann Neurol 2007

It is well established that the major histocompatibility complex (MHC) on chromosome 6p21 contains at least one gene that influences susceptibility to multiple sclerosis.^1^–^6^ Although this association was first identified more than 30 years ago^1^ through the study of class I human leukocyte antigens (HLAs), it was quickly realized that this signal was predominantly, if not exclusively, the result of linkage disequilibrium (LD) with class II HLA genes, and that these exert the primary effect on susceptibility.^7^, ^8^ The complex nature of the MHC, especially its high gene content, extreme polymorphism, and extensive LD,^9^ has confounded efforts to resolve the nature of the MHC association in multiple sclerosis, although progress and useful clarifications have been made, especially in recent years.

In virtually every population studied, multiple sclerosis is found to be associated with the DRB1*1501 allele.^10^ The only exceptions are those populations where this allele has a low frequency, and analysis is therefore underpowered; but even in these situations, DRB1*1501 is generally overrepresented in cases.^11^ The DRB1*1501 allele is carried on a particularly extensive haplotype,^12^ the most common *DR15* haplotype found in white Europeans. As a result, many variants from flanking genes, even some located quite a distance from *DRB1*, have sufficient LD with DRB1*1501 that they invariably also show evidence for association with the disease in any population where association with DRB1*1501 can be demonstrated.^11^, ^13^–^17^ This extensive LD has made it difficult to establish which of the variants making up this haplotype is primarily responsible for the association. The distinction between DRB1*1501 and DQB1*0602 has been particularly taxing because the LD between these closely mapped genes is especially tight in those populations where the disease is frequent, that is, white Europeans and their migrant descendants. However, recent studies in the admixed African American population indicate the supremacy of the DRB1*1501 allele.^18^

In the presence of one susceptibility allele it is difficult to identify effects attributable to a second allele,^19^ especially if the second allele exerts a more modest effect or has a low frequency, or both. However, by analyzing populations where *DR15* haplotypes are less common, and by using large cohorts, it has been possible to demonstrate that the DRB1*0301 allele also confers susceptibility to multiple sclerosis, thereby confirming allelic heterogeneity at the *DRB1* locus.^4^, ^6^, ^18^, ^20^ Furthermore available evidence suggests that the susceptibility effects of the DRB1*1501 allele may be modulated by other *DRB1* alleles.^6^, ^20^

The relation between the MHC and multiple sclerosis is further complicated by the accumulating evidence suggesting that MHC loci mapping outside *DRB1* also influence susceptibility to the disease.^11^, ^13^–^16^ Work in animal models suggests that clustering of susceptibility loci is a common phenomenon in complex disease,^21^ and it therefore appears reasonable to expect that other genes from the MHC region may influence susceptibility to multiple sclerosis. The observation of positive logarithm of odds scores in the MHC region in linkage studies stratified for the effects of *DRB1* supports the existence of secondary loci,^22^–^24^ although none of these data reaches a level providing statistical confidence. In considering these linkage data, it is important to remember that early linkage studies in multiple sclerosis^25^–^27^ were significantly underpowered,^28^ to the point that they could not even convincingly demonstrate evidence for linkage resulting from the effects of *DRB1*. Confirmation of linkage in this region has been established only in more recent studies involving many hundreds of families.^23^, ^24^, ^29^ Given the inherently limited resolution of linkage-based studies,^28^ the absence of statistically significant linkage in the MHC region after exclusion of primary effects attributable to *DRB1* does not exclude the presence of secondary loci. Several authors have attempted to identify secondary loci using more powerful association-based methods. Two groups have typed dense microsatellite maps of the region and both found evidence for a secondary locus maximal in a region close to *HLA*-*A*: one group identifying the marker D6S1683 just telomeric of *HLA*-*A*,^11^ and the second group implicating a region including *HLA*-*A* extending from MOGCA to D6S265 marker.^14^ Follow-up studies in Norway also found evidence implicating the D6S265 marker.^16^ In another smaller study, a microsatellite marker close to *HLA*-*C* (marker C1_3_2) also showed evidence for an independent effect.^15^ In contrast, a systematic effort to screen the MHC and flanking regions using single nucleotide polymorphisms (SNPs) found no evidence for association beyond that attributable to DRB1*1501, although this study was limited by a high genotyping failure rate (40%) and, more importantly, a distribution of markers leaving regions close to the classical loci essentially unexplored.^17^ In all of these studies, statistical power has inevitably been reduced by the processes required to filter out the primary effect attributable to *DRB1* and the large correction required for multiple testing. Unfortunately, none of the published studies has used sufficient samples to compensate for these statistical penalties, and thus none is able to provide unequivocal evidence supporting any particular secondary locus.

## Subjects and Methods

### UK Trio Families and Sporadic Cases

The 480 trio families (an affected individual and both parents) and 721 sporadic cases participating in our study were recruited from across the United Kingdom. All subjects involved in this study gave written informed consent and provided a venous blood sample from which DNA was extracted and normalized. Comparing data from the five classical loci in the trio family index cases (n = 480) with those from the sporadic cases (n = 721) showed that there was no statistically significant difference between these two cohorts. The clinical details for each set of cases are summarized in Table 1. All cases were diagnosed according to recognized criteria.^30^, ^31^

**1:** Patient Demographics

Demographics	US Trio Index (n = 450)	UK Trio Index (n = 480)	UK Sporadic (n = 721)
Sex (M:F)	1:3.2	1:3.2	1:2.5
Mean age (yr)	39	38	48
Mean age at onset (yr)	29	25	33
Mean EDSS	4.0	4.4	4.6
Mean duration (yr)	11	13	15

The slightly younger age and greater proportion of female individuals seen in cases from the trio families in each population reflects the requirement for both parents to be alive and willing to take part. This necessarily means that these patients tend to be younger, and because the disease has a younger age at onset in female individuals, also results in an increased proportion of women.

EDSS = Extended Disability Status Scale.

### UK Extension Analysis Control Cohorts

The fully anonymous control data used in the extension analysis were derived from three sources: local organ donors, national organ donor records held by UK Transplant (UKT), and the 1958 birth cohort. Ethical permission for using these data was obtained from the appropriate respective research ethics committees. Data from all five classical loci (*HLA*-*A*, -*B*, -*C*, -*DRB1*, and -*DQB1*) were available for the donor and UKT cohorts, whereas these were available for only three loci in the 1958 birth cohort (*HLA*-*B*, -*DRB1*, and -*DQB1*). Only white individuals from these cohorts with complete data were included: 408 for the donor cohort, 2,201 for the UKT cohort, and 1,051 for the 1958 birth cohort (total 3660 individuals). There was no evidence for any statistically significant difference among these three cohorts in a pairwise comparison of the classical loci. We also compared each of the three control cohorts with the nontransmitted alleles from the 480 trio families. Again, there was no evidence for any statistically significant difference.

### US Trio Families

All cases from the 450 US trio families were diagnosed according to the McDonald criteria.^31^ All individuals involved in this study gave written informed consent using documents approved by the institutional review board and provided a venous blood sample from which DNA was extracted and normalized. The clinical details for the 450 index cases from the trio families are summarized in Table 1.

### Screening Single Nucleotide Polymorphisms

The recently completed resequencing of the MHC region from consanguineous homozygous cell lines carrying specific disease-associated haplotypes provided a comprehensive and detailed description of these haplotypes.^32^, ^33^ By comparing the sequence from the PGF line, which carries the multiple sclerosis–associated DR15 haplotype (HLA-A3-B7-Cw7-DR15), with the other completed haplotypes, COX (HLA-A1-B8-Cw7-DR3) and QBL (A26-B18-Cw5-DR3), we were able to identify 241 coding variants (outside the hypervariable regions). These variants distinguish the multiple sclerosis–associated haplotype from alternatives and are therefore especially promising candidate susceptibility variants. To increase the coverage provided by this set of markers, we also developed assays for SNPs already identified as tagging common haplotypes in the MHC region^34^ and supplemented this list with variants from the class III region. Working assays were established for a total of 110 SNPs, including 5 from the extended class I region, 1 from the extended class II region, and 104 from the classical MHC. Seventy SNPs were genotyped using a Sequenom MassArray MALDI-TOF platform,^35^ whereas the remaining 40 were genotyped using TaqMan allelic discrimination assays on an ABI7900HT genotyping platform (Applied Biosystems, Foster City, CA).^36^ The primer sequences used and basic performance characteristics for each marker are included in Supplementary Table S1.

### Screening Microsatellites

To generate a screening set of microsatellites, we first identified an exhaustive list of such markers (n = 248) lying within the extended MHC using published^37^–^41^ and publicly available resources: National Center for Biotechnology Information (NCBI) UniSTS (Build 34.3; http://www.ncbi.nlm.nih.gov/entrez/query.fcgi?db=unists), GDB Human Genome Database (http://www.gdb.org), and Ensembl (Build 22.34d.1; http://www.ensembl.org/index.html). From Ensembl BLAST (http://www.ensembl.org/Multi/blastview) and electronic polymerase chain reaction (ePCR) analysis^42^ (NCBI; http://www.ncbi.nlm.nih.gov/sutils/e-pcr), with emphasis on markers previously suggested to be of relevance in multiple sclerosis,^11^, ^14^, ^15^ we then selected markers from this list to produce an informative map with a density of approximately 1 marker per 50 to 100kb across the classical MHC region (29.8–33.2Mb^9^). The 69 markers selected were then typed in a trial set of 122 trio families. Seventeen markers were found to be monomorphic and two assays failed; we used the remaining 50 markers in our study. These 50 included 8 from the extended class I region, 4 from the extended class II region, and 38 from the classical MHC itself. Each microsatellite was amplified by PCR using TrueAllele PCR Premix and the manufacturer's standard conditions (Applied Biosystems). The PCR products were genotyped on a 3700 Genetic Analyzer (Applied Biosystems) using GENESCAN version 3.5 (Applied Biosystems) and GENOTYPER version 3.7 (Applied Biosystems) software. The primer sequences used and basic performance characteristics for each marker are included in Supplementary Table S1.

### Human Leukocyte Antigen Typing

In the screening of trio families, four-digit (medium-resolution) typing of *HLA*-*DRB1* and *HLA*-*DQB1* was performed in all the UK trios and 60% of the US trios.^20^, ^43^ In the remaining US trios, lower resolution typing was performed as described later. For the analysis of all 930 trio families together, the resolution was down grouped to the low-resolution level. The class I loci in the UK trio families and all the HLA typing performed in the sporadic cases was low-resolution typing based on PCR with sequence-specific primers, as developed by Olerup and Zetterquist^44^ and previously used by us.^45^ Alleles were assigned using World Health Organization nomenclature. The *HLA*-DRB1*15 alleles were subtyped into *HLA*-DRB1*1501, *1502*, or *1503*. The primer sequences and combinations for each PCR reaction are given in Supplementary Table S2.

LD between *DRB1* and the other classical loci was confirmed in both the trio family and extension data sets; estimates for Global D' and Cramer's V (measures of LD between multiallelic loci) are summarized in Table 2. As expected, the extent of LD is inversely correlated with the distance from *DRB1*. It should be noted that although the extent of LD between class I loci and *DRB1* is modest, this level of LD is sufficient to produce an association signal at class I loci secondary to *DRB1* effects. Indeed, this is precisely how the association between the MHC and multiple sclerosis was first recognized. Allele counts and frequencies for the five classic loci in the screening and extension data sets are provided in Supplementary Table S3.

**2:** Multiallelic Measures of Linkage Disequilibrium between DRB1 and the Other Classical Loci

Locus	Cases	Control Subjects	Transmitted	Nontransmitted
Global D'				
*HLA*-*A*	0.24	0.24	0.29	0.32
*HLA*-*C*	0.42	0.44	0.51	0.53
*HLA*-*B*	0.49	0.47	0.56	0.56
*HLA*-*DQB1*	0.91	0.91	0.97	0.95
Cramer's V				
*HLA*-*A*	0.18	0.19	0.27	0.28
*HLA*-*C*	0.26	0.28	0.31	0.36
*HLA*-*B*	0.34	0.36	0.31	0.33
*HLA*-*DQB1*	0.78	0.79	0.82	0.82

The higher resolution of *HLA*-*DRB1* and *HLA*-*DQB1* typing together with the greater degree of phase information explains why the estimates for linkage disequilibrium are generally higher in this cohort than in the case–control analysis.

### Statistical Analysis

Transmission disequilibrium testing (TDT) of data from trio families was performed using the TDTPHASE program, part of the UNPHASED suite.^46^ Before analysis, Mendelian errors were zeroed out using the PEDCHECK program,^47^ and basic performance characteristics were established using the PEDSTATS program.^48^ In the extension study, case–control association testing was performed using the COCAPHASE program, also part of the UNPHASED suite.^46^ In all tests, we used the EM option and grouped rare alleles (haplotypes) with expected counts of less than 10 in both case (transmitted) and control subjects (nontransmitted). This cutoff was chosen to ensure that we did not include alleles where the available data were insufficient to provide any power. In conditional analysis, we used the main-effects test. Measures of LD were also calculated using the relevant UNPHASED program. For multiallelic markers, UNPHASED calculates Global D' and Cramer's V as measures of the overall extent of LD. Nontransmitted alleles in the trio families were identified using the MERLIN program,^49^ ignoring alleles where phase was uncertain.

## Results

To refine the relation between the MHC and multiple sclerosis, we first screened the region by genotyping 110 SNPs and two class II HLA loci (*HLA*-*DRB1* and *HLA*-*DQB1*) in a cohort of 930 trio families (an affected individual and their parents): 480 from the United Kingdom and 450 from the United States. In addition, we also typed 50 microsatellite markers and 3 class I HLA loci (*HLA*-*A*, -*B*, and -*C*) in the same 480 UK trios. As expected, multiple markers showed highly significant evidence for association (see Supplementary Table S4 for individual results). To eliminate the confounding effects of LD with the DRB1*1501 allele, we excluded all families where either parent carried a DRB1*1501 allele and reanalyzed the data from the remaining 318 families (146 from the United Kingdom and 172 from the United States). The results generated in this subgroup analysis are also listed in Supplementary Table S4. After stringent Bonferroni correction for the number of markers tested (n = 165), we found that two markers continued to show statistically significant evidence for association: *HLA*-*C* (*p*_corrected_ = 0.04) and rs3132552 (*p*_corrected_ = 0.006), a synonymous coding polymorphism in the corneodesmosin gene. Because rs3132552 lies just 151kb telomeric of *HLA*-*C*, it was not surprising to find substantial LD between these loci in the UK trios (Global D' = 0.61). Testing rs3132552 in the DRB1*1501-negative trios from the United States and United Kingdom independently demonstrated that the marker shows association in both populations (*p*_uncorrected_ = 0.001 in the US cohort; *p*_uncorrected_ = 0.009 in the UK cohort). These data demonstrate that there is significant residual association within the MHC region above and beyond that attributable to the well-established association with DRB1*1501. However, the power available in this modest DRB1*1501-negative subgroup, together with the extensive LD between the various MHC loci, makes it impossible to establish with any confidence which locus is primarily responsible for the observed effect.

Consequently, to further refine the nature of the secondary MHC association identified in our screening experiment, we typed the five classical HLA loci (*HLA*-*A*, -*B*, -*C*, -*DRB1*, and -*DQB1*) in an additional 721 sporadic UK multiple sclerosis patients and established UK control data from a cohort of 3,660 individuals (see Subjects and Methods). Given that the US trio families provided independent evidence implicating the HLA-C region, we analyzed these new data from additional UK samples together with those from the 480 UK index cases used in the first screening experiment in the form of an extension analysis as opposed to a replication study.^50^ Results from the first-pass unstratified analysis of all 1,201 cases and 3,660 control subjects are shown in Table 3. Association with *DRB1* is overwhelmingly the most significant, with the majority of this effect attributable to the DRB1*1501 allele (*p*_uncorrected_ = 4.5 × 10^−88^). After excluding all individuals carrying DRB1*1501, analysis of the remaining data continues to show highly significant evidence for association with the most significant effect still appearing to come from the *DRB1* locus (see Table 3). As expected, the majority of the residual *DRB1* effect is attributable to the DRB1*03 allele (*p*_uncorrected_ = 6.3 × 10^−5^). Therefore, we next excluded all individuals carrying DRB1*03 alleles. Analysis of the remaining data shows that significant evidence for association is still evident but only at *HLA*-*C* and *DRB1* (see Table 3). At *DRB1*, the only allele showing significant evidence for association is the DRB1*0103 allele (*p*_uncorrected_ = 1.8 × 10^−5^). Even though this allele is relatively uncommon, we also excluded all individuals carrying the DRB1*0103 allele. In this final stratified analysis, statistically significant association is only apparent at the *HLA*-*C* locus (see Table 3). Analysis of *HLA*-*C* after conditioning on *DRB1* has no important effect on the evidence for association at this locus, whereas conditioning on *HLA*-*C* confirms that none of the four other loci exerts any residual main effects.^19^, ^51^ Inspection of the individual *HLA*-*C* alleles in the extension analysis shows that the *HLA*-C*05 allele is the most significantly associated, being underrepresented in cases (Table 4). Following a replication approach, in which the original 480 index cases from the trio families are excluded, has little effect on the interpretation of the results. The results from this replication approach are shown in the lower half of Table 3 for comparison. Our analysis indicates that three *DRB1* alleles (*1501, *03, and *0103) and one *HLA*-*C* allele (*05) exert independent effects on susceptibility to multiple sclerosis. To determine the risk associated with each *DRB1* susceptibility allele in haplotypes with and without *HLA*-C*05, we reanalyzed the full data set three times, first excluding all individuals carrying DRB1*03 or *0103, next excluding all individuals carrying DRB1*1501 or *0103, and then finally after excluding all individuals carrying DRB1*1501 or *03. Table 5 shows the relative risk associated with the various haplotypic combinations of *DRB1* and *HLA*-*C* alleles, as compared with haplotypes carrying no associated allele at either locus. The relative risk associated with haplotypes carrying *HLA*-C*05 without *DRB1* risk alleles is consistent across the three analyses and is significantly less than 1 in each case, confirming the protective nature of this allele. The data suggest that the risk associated with the DRB1*1501 allele is reduced but not abolished by the presence of an *HLA*-C*05 allele on the same haplotype. Unfortunately, the frequency of the other combined haplotypes (DRB1*03 with *HLA*-C*05 and DRB1*0103 with *HLA*-C*05) was too low to provide sufficient statistical power to make a judgment about whether the *HLA*-C*05 allele alters the risk associated with the secondary *DRB1* risk alleles.

**3:** Association of Classical Human Leukocyte Antigen Loci in the Case–Control Data Sets

Cohort Subgroups	*HLA*-*A*	*HLA*-*C*	*HLA*-*B*	*HLA*-*DRB1*	*HLA*-*DQB1*	Cases, N	Control Subjects, N	Power, %^a^
Extension Analysis								
Full	**3.3** × **10**^−**7**^	**1.4** × **10**^−**16**^	**4.3** × **10**^−**28**^	**9.1** × **10**^−**94**^	**1.2** × **10**^−**21**^	1,201	3,660	99.9
DRB1*15 excluded	**3.4** × **10**^−**4**^	**3.2** × **10**^−**5**^	**5.3** × **10**^−**6**^	**5.2** × **10**^−**7**^	**3.1** × **10**^−**4**^	481	2,635	95.2
DRB1*15 and *03 excluded	0.042	**2.3** × **10**^−**5**^	0.017	**2.9** × **10**^−**4**^	0.016	297	1,802	75.8
DRB1*15, *03, and *0103 excluded	0.053	**5.9** × **10**^−**5**^	0.014	0.18	0.0075	264	1,724	69.5
Replication analysis								
Full	**1.1** × **10**^−**4**^	**1.2** × **10**^−**10**^	**7.9** × **10**^−**13**^	**6.7** × **10**^−**53**^	**4.2** × **10**^−**14**^	1,201	3,660	99.6
DRB1*15 excluded	5.4 × 10^−3^	**1.5** × **10**^−**3**^	**6.3** × **10**^−**4**^	**9.7** × **10**^−**4**^	**9.6** × **10**^−**4**^	481	2,635	79.8
DRB1*15 and *03 excluded	0.21	**2.6** × **10**^−**4**^	0.010	0.16	0.012	297	1,802	50.2
DRB1*15, *03, and *0103 excluded	0.36	**1.2** × **10**^−**3**^	0.017	0.61	8.2 × 10^−3^	264	1,724	44.9

As each of the 5 markers has been tested 4 times in each approach, a Bonferroni correction factor of no more than 20 is required. Applying this to the nominal *p values* included in the table indicates that only those in bold are significant after this conservative correction for multiple testing.

This column indicates the power of each analysis to identify a common allele (frequency 10%) conferring a risk with an odds ratio of 1.6 under a multiplicative model at a level of significance sufficient to survive Bonferroni correction (nominal *p* = 0.0025).^57^

**4:** Individual HLA-Cw Allele Associations in Final Subgroup of Data (in Which All Individuals Carrying DRB1*1*15, *03, and *0103 alleles have been excluded; see Table 3)

Allele	Cases, N (%)	Control Subjects, N (%)	*p*
01	12 (2)	116 (5)	0.0061
02	34 (7)	133 (6)	0.38
03	76 (15)	403 (17)	0.22
04	67 (13)	252 (10)	0.12
05	37 (7)	320 (13)	3.3 × 10^−5^
06	71 (14)	278 (12)	0.19
07	110 (21)	470 (20)	0.41
08	32 (6)	113 (5)	0.18
12	18 (3)	75 (3)	0.69
14	6 (1)	21 (1)	0.56
15	24 (5)	49 (2)	0.0017
16	29 (6)	153 (6)	0.49
17	3 (1)	18 (1)	0.66

**5:** Haplotype Analysis of HLA-C and HLA-DRB1 in Subsets of the Case–Control (Extension) Cohort Showing the Relative Risk with 95% Confidence Intervals for Each Haplotype

*HLA*-*C*	*HLA*-*DRB1*	Cases, N (%)	Control Subjects, N (%)	RR	CI
Analysis 1 (excluding all individuals carrying DRB1*03 or *0103)					
*R^a^	*X^a^	900.3 (54.7)	2,589.0 (70.3)	1.00	—
*05	*X	75.7 (4.6)	395.7 (10.7)	0.55	0.40–0.76
*R	*1501	634.7 (38.6)	652.7 (17.7)	2.80	2.42–3.23
*05	*1501	35.3 (2.1)	46.3 (1.3)	2.19	1.18–4.10
Analysis 2 (excluding all individuals carrying DRB1*1501 or *0103)					
*R^a^	*Y^a^	611.1 (70.1)	2,521.0 (70.8)	1.00	—
*05	*Y	50.9 (5.8)	396.5 (11.1)	0.53	0.37–0.75
*R	*03	182.9 (21.0)	579.5 (16.3)	1.30	1.08–1.57
*05	*03	27.2 (3.1)	64.5 (1.8)	1.74	0.96–3.15
Analysis 3 (excluding all individuals carrying DRB1*1501 or *03)					
*R^a^	*Z^a^	514.0 (87.7)	2,121.0 (84.7)	1.00	—
*05	*Z	39.0 (6.7)	332.0 (13.3)	0.49	0.34–0.69
*R	*0103	33.0 (5.6)	51.0 (2.0)	2.96	1.63–4.37
*05	*0103	≈0 (≈0.0)	≈0 (≈0.0)	—	—

*R indicates any *HLA*-*C* allele except *05, *X any *DRB1* allele except *1501, *Y any *DRB1* allele except *03, and *Z any *DRB1* allele except *0103.

RR = relative risk; CI = confidence interval.

## Discussion

Using a combination of microsatellite, SNP, and HLA typing in family-based and case–control cohorts from two different populations, we have shown that *HLA*-*C* exerts an independent effect on susceptibility to multiple sclerosis above and beyond any effects attributable to the nearby *DRB1* gene. We found no support for effects attributable to *HLA*-*A* or any of the microsatellite loci previously suggested by other researchers, although such effects cannot be excluded at this time.^11^, ^13^, ^14^, ^16^ It remains possible that the observed association with *HLA*-*C* is secondary to LD with a nearby but as yet untyped variant. The absence of any residual main effects at *DQB1*, *HLA*-*B*, and *HLA*-*A* make it unlikely that the observed association results from these loci despite their strong LD with *HLA*-*C*. As with all genetic analyses of complex diseases, our study has a number of limitations that need to be considered when evaluating its conclusions.

Adequate correction for multiple testing is particularly important in studies that involve systematic screening and stratification because the number of tests performed is generally large.^52^ However, calculating an appropriate correction factor can be difficult when there is LD between markers or when subsets (strata) of data are analyzed in addition to total data sets. In these situations, tests are partially correlated rather than fully independent, and simply counting the number of tests performed provides an excessively conservative correction factor. Application of such a crude Bonferroni correction^53^ runs the risk for inflating the type II error rate unless the sample size used is sufficient to compensate for this conservatism. However, inaccurate attempts to assess the degree of interdependence between tests might underestimate the correction required, thereby resulting in a type I error. Because these plague the genetic analysis of multiple sclerosis, we elected to apply the conservative Bonferroni corrections at each stage. It might be argued that even this approach is insufficient and that we should account for all of the tests performed across the whole study and not just those used at each stage. The evidence for association we identified concerning *HLA*-*C* would remain significant even if such a project-wide correction factor (approximately 300) were to be applied.

Association testing can be confounded by population stratification and other phenomena that lead to inadequate matching of cases and control subjects. Our use of trio families and transmission disequilibrium testing protects against these confounders in the screening phase. However, the extension phase experiments used case–control analysis; therefore, results emerging from these efforts could have been confounded by such errors. The classical loci are highly polymorphic and provide a powerful means to test for stratification. The absence of any difference between the three UK control cohorts or the two sets of UK cases is therefore extremely reassuring in this respect. Perhaps even more reassuring is the absence of any difference between the unrelated UK control cohorts and nontransmitted alleles from the UK trio families, suggesting that our cases are drawn from the same genetic background as the control subjects and making it unlikely that hidden population stratification accounts for our observations. However, replication of the findings in independent cohorts will be necessary to fully exclude this possibility.

Efforts to further replicate our findings will require considerable resources because exclusion of individuals carrying *DRB1* susceptibility alleles means that only 47% of control subjects and 22% of cases will ultimately be informative for the study of *HLA*-*C*. Large initial cohorts would need to be selected to provide realistic power. The absence of any statistically significant difference in an underpowered study should not be misinterpreted as evidence against this effect. Just as it proved difficult to establish that DRB1*1501 is responsible for the primary effect in this region, it may prove even more difficult to refine this secondary effect in detail. It remains possible that the observed association is secondary to LD with a nearby but as yet untyped variant. The absence of any residual main effects at *DQB1*, *HLA*-*B*, and *HLA*-*A* make it unlikely that the observed association results from these loci despite their strong LD with *HLA*-*C*.

If *HLA*-*C* is, in fact, the locus primarily responsible for our observations, this would implicate novel pathways in disease pathogenesis, in particular, the innate immune system. HLA-C molecules, loaded with nonamer peptides, act as ligands for the killer cell immunoglobulin-like receptors (KIRs). KIR receptors contain two or three immunoglobulin-like domains and a short (*KIR2DS*, *KIR3DS*) or long (*KIR2DL*, *KIR3DL*) cytoplasmic tail, corresponding to an activating or inhibitory action on the natural killer cells and T-cell subsets on which they are expressed. Whereas the ligands for activating KIRs remain elusive, and may have relatively weak binding affinities, those for the inhibitory KIRs are well defined. The subset of *HLA*-*C* alleles with Ser at position 77 and Asn at position 80 (C1 group) bind to the *KIR2DL2* and *KIR2DL3* receptors, whereas those with Asn at position 77 and Lys at position 80 (C2 group) bind to the *KIR2DL1* receptor. *HLA*-*C* peptide binding specificity may further influence interaction with KIRs.^54^ No evidence for association was seen in our data after grouping *HLA*-*C* alleles according to this functional categorization, suggesting that the protective effect seen for *HLA*-C*05 is specific to this allele, and not a consequence of its group function. Alternately, as the effect appears to be allele rather than functional group specific, it may indicate that susceptibility to multiple sclerosis is conferred by an aspect of *HLA*-*C* function that is independent of its interaction with KIR.

The established association of *HLA*-C*06 with susceptibility to psoriasis provides a clear precedent for the involvement of *HLA*-*C* in complex inflammatory disease. In a Sardinian study of psoriasis, *HLA*-C*05 was found to be significantly underrepresented (protective) in patients, although it was not established whether this effect is independent of or secondary to the overrepresentation of *HLA*-C*06 and/or haplotypes containing risk alleles at the nearby corneodesmosin (CDSN) gene,^55^ in which our most highly associated SNP, rs3132552, is found. Recently, the presence of the activating *KIR2DS1* and *KIR2DS2* genes was reported to be a novel risk factor for psoriasis and an interaction between *HLA*-*C* and KIR observed with the overall combination of activating and inhibitory genotypes influencing susceptibility.^56^ Association of combinations between HLA class I and *KIR* genes has also been reported for a range of other infectious and autoimmune diseases.^54^ This provides a strong rationale for investigating the *KIR* gene cluster as a candidate susceptibility locus in multiple sclerosis subsequent to our finding of association with *HLA*-*C*. The fact that the *KIR* gene cluster lies on chromosome 19 where modest evidence of linkage was observed in the recent high-density screen for linkage in multiple sclerosis^24^ lends further support. In concordance with these results, this chromosome 19 linkage signal only declares itself in an ordered subset analysis based on those families not showing linkage at MHC, essentially those where the effects of DRB1*1501 have been excluded.^24^

In conclusion, we show that the class I gene *HLA*-*C*, or a locus in tight LD with it, confers additional effects on susceptibility to multiple sclerosis, substantially adding to our understanding of the MHC region in this disease and offering a clear roadmap to further experiments that will refine these observations in larger data sets.
