# Supplementary material for: A second major histocompatibility complex susceptibility locus for multiple sclerosis
Source: Ann Neurol. 2007 Mar;61(3):228–36. doi: 10.1002/ana.21063 (PMC2737610; doi:10.1002/ana.21063)
Supplement: Supplementary file 2 [file ana0061-0228-SD2.doc]

# Table S2 HLA SSP PCR Primer Sequences and Reactions Combinations

| **Primer** | **Sequence** | **Present in Reactions^a^** |
| --- | --- | --- |
| Cont63 | TGCCAAGTGGAGCACCCAA | Internal Positive Control: HLA-A^b^, -B^b^, -Cw^b^ reactions |
| Cont64 | GCATCTTGCTCTGTGCAGAT | Internal Positive Control: HLA-A^b^, -B^b^, -Cw^b^ reactions |
| 3'HGH | TCACGGATTTCTGTTGTGTTT | Internal Positive Control: HLA-DRB1^b^, HLA-DRB1*15 ^b^, -DQB1^b^ reactions |
| 5'HGH | GCCTTCCCAACCATTCCCTTA | Internal Positive Control: HLA-DRB1^b^, HLA-DRB1*15 ^b^, -DQB1^b^ reactions |
| 5-A1 | CGACGCCGCGAGCCAGAA | HLA-A-Rxn 1, 4 |
| 5-A2 | GTGGATAGAGCAGGAGGGT | HLA-A-Rxn 2 |
| 5-A3 | AGCGACGCCGCGAGCCA | HLA-A-Rxn 3 |
| 5-A4 | TATTGGGACGAGGAGACAG | HLA-A-Rxn 5 |
| 5-A5 | GGCCGGAGTATTGGGACGA | HLA-A-Rxn 6, 7 |
| 5-A6 | GGGTACCAGCAGGACGCT | HLA-A-Rxn 8, 13 |
| 5-A7 | GGAGTATTGGGACCGGAAC | HLA-A-Rxn 9 |
| 5-A8 | TCACAGACTGACCGAGAGAG | HLA-A-Rxn 10, 20 |
| 5-A9 | TCACAGACTGACCGAGCGAA | HLA-A-Rxn 11 |
| 5-A10 | CCGGAGTATTGGGACCTGC | HLA-A-Rxn 12, 17 |
| 5-A11 | ACGGAATGTGAAGGCCCAG | HLA-A-Rxn 14, 15, 16, 23, 24 |
| 5-A12 | CCCGGCCCGGCAGTGGA | HLA-A-Rxn 18 |
| 5-A13 | GATAGAGCAGGAGAGGCCT | HLA-A-Rxn 19^c^ |
| 5-A14 | CCACTCCATGAGGTATTTCTT | HLA-A-Rxn 22^e^ |
| 5-A15 | CCACTCCATGAGGTATTTCAC | HLA-A-Rxn 21^d^ |
| 5-A16 | TACTACAACCAGAGCGAGGA | HLA-A-Rxn 25 |
| 5-A17 | GGGTACCGGCAGGACGCT | HLA-A-Rxn 13 |
| 5-A18 | TGGCCCTGACCCAGACCA | HLA-A-Rxn 26 |
| 3-A1 | AGCCCGTCCACGCACCG | HLA-A-Rxn 1 |
| 3-A2 | CCAAGAGCGCAGGTCCTCT | HLA-A-Rxn 2, 5, 23 |
| 3-A3 | CACTCCACGCACGTGCCA | HLA-A-Rxn 3 |
| 3-A4 | GAGCCACTCCACGCACGT | HLA-A-Rxn 4, 13 |
| 3-A5 | CCTCCAGGTAGGCTCTCAA | HLA-A-Rxn 6 |
| 3-A6 | CCTCCAGGTAGGCTCTCTG | HLA-A-Rxn 7, 16 |
| 3-A7 | GAGCCACTCCACGCACCG | HLA-A-Rxn 8, 15 |
| 3-A8 | GAGCCACTCCACGCACTC | HLA-A-Rxn 8 |
| 3-A9 | ATGTAATCCTTGCCGTCGTAA | HLA-A-Rxn 9, 10, 11, 12, 14 |
| 3-A10 | AGCGCAGGTCCTCGTTCAA | HLA-A-Rxn 17, 19 ^c^, 20, 22, 26 |
| 3-A11 | CCGTCGTAGGCGTGCTGT | HLA-A-Rxn 18 |
| 3-A13 | GCCTTCACATTCCGTGTGTT | HLA-A-Rxn 21 |
| 3-A14 | CCGCGGAGGAAGCGCCA | HLA-A-Rxn 24 |
| 3-A15 | GAGCCCGTCCACGCACTC | HLA-A-Rxn 25 |
| 5-B5 | CGAGAGAGCCTGCGGAAC | HLA-B-Rxn 26 |
| 5-B7 | GGAGTATTGGGACCGGAAC | HLA-B-Rxn 1, 46, 47^c^, 51^c^ |
| 5-B9 | GACCGGAACACACAGATCTT | HLA-B-Rxn 2^c^, 3, 48 |
| 5-B10 | GCTACGTGGACGACACGCT | HLA-B-Rxn 4, 13, 29 |
| 5-B11 | ACCGAGAGAACCTGCGGAT | HLA-B-Rxn 5^c^, 19, 21^c^, 50 |
| 5-B12 | CCACTCCATGAGGTATTTCC | HLA-B-Rxn 6^c^ |
| 5-B13 | CCGAGAGAGCCTGCGGAA | HLA-B-Rxn 7, 17, 49^d^ |
| 5-B14 | GGGGAGCCCCGCTTCATT | HLA-B-Rxn 8 |
| 5-B15 | CGCCACGAGTCCGAGGAA | HLA-B-Rxn 8, 10, 12 |
| 5-B16 | ACCGGGAGACACAGATCTC | HLA-B-Rxn 9, 11, 31, 32, 40, 52 |
| 5-B17 | CGCGAGTCCGAGGATGGC | HLA-B-Rxn 14^c^, 39, 45 |
| 5-B18 | AGCAGGAGGGGCCGGAA | HLA-B-Rxn 15 |
| 5-B19 | CCATGAGGTATTTCTACACCG | HLA-B-Rxn 16^c^, 38 |
| 5-B20 | GACCGGAACACACAGATCTA | HLA-B-Rxn 18 |
| 5-B21 | TACCGAGAGAACCTGCGC | HLA-B-Rxn 19 |
| 5-B22 | CGCCGCGAGTCCGAGAGA | HLA-B-Rxn 20, 25^c^, 35, 42 |
| 5-B23 | AACATGAAGGCCTCCGCG | HLA-B-Rxn 22, 23, 36 |
| 5-B24 | GCGCCGTGGATAGAGCAA | HLA-B-Rxn 24 |
| 5-B25 | CAGATCTACAAGGCCCAGG | HLA-B-Rxn 27^c^, 34 |
| 5-B26 | GCCGCGAGTCCGAGAGG | HLA-B-Rxn 28^c^ |
| 5-B27 | GCCGCGAGTCCGAGGAC | HLA-B-Rxn 30 |
| 5-B28 | TACTACAACCAGAGCGAGGA | HLA-B-Rxn 33^c^ |
| 5-B29 | GAGACACAGAAGTACAAGCG | HLA-B-Rxn 37^c^ |
| 5-B30 | GGGAGCCCCGCTTCATCT | HLA-B-Rxn 41, 43^c^ |
| 5-B31 | ACCGGAACACACAGATCTG | HLA-B-Rxn 44 |
| 5-B32 | GACGACACCCAGTTCGTGA | HLA-B-Rxn 53, 54, 55 |
| 5-B33 | GACGACACGCTGTTCGTGA | HLA-B-Rxn 53, 54, 55 |
| 5-B34 | GACGACACGCAGTTCGTGA | HLA-B-Rxn 53, 54, 55 |
| 3-B2 | TACCAGCGCGCTCCAGCT | HLA-B-Rxn 1 |
| 3-B3 | CCTCCAGGTAGGCTCTGTC | HLA-B-Rxn 2^c^, 30 |
| 3-B4 | CCGCGCGCTCCAGCGTG | HLA-B-Rxn 3, 9, 34 |
| 3-B5 | CTCTCAGCTGCTCCGCCT | HLA-B-Rxn 4, 36 |
| 3-B6 | ATCCTTGCCGTCGTAGGCT | HLA-B-Rxn 5^c^, 6^c^, 14^c^, 26 |
| 3-B7 | CCAGGTATCTGCGGAGCG | HLA-B-Rxn 7, 45 |
| 3-B8 | GTCGTAGGCGTCCTGGTC | HLA-B-Rxn 8 |
| 3-B9 | CGTCGTAGGCGTACTGGTC | HLA-B-Rxn 8 |
| 3-B10 | TCCCACTTGCGCTGGGT | HLA-B-Rxn 10 |
| 3-B11 | GCGGCGGTCCAGGAGCG | HLA-B-Rxn 11 |
| 3-B12 | GAGCCACTCCACGCACTC | HLA-B-Rxn 12 |
| 3-B13 | CTCCAACTTGCGCTGGGA | HLA-B-Rxn 13, 35 |
| 3-B14 | CGTCGCAGCCATACATCCA | HLA-B-Rxn 15 |
| 3-B15 | GTGTGTTCCGGTCCCAATAT | HLA-B-Rxn 16^c^ |
| 3-B16 | CGTGCCCTCCAGGTAGGT | HLA-B-Rxn 17, 18, 19, 20 |
| 3-B17 | GCCATACATCCTCTGGATGA | HLA-B-Rxn 21^c^, 41, 47^c^ |
| 3-B18 | CGTCGCAGCCATACATCAC | HLA-B-Rxn 22 |
| 3-B19 | GAGGAGGCGCCCGTCG | HLA-B-Rxn 23, 48 |
| 3-B20 | CTTGCCGTCGTAGGCGG | HLA-B-Rxn 24, 40, 42 |
| 3-B21 | CCATACATCGTCTGCCAA | HLA-B-Rxn 25^e^, 28^c^, 33^e^ |
| 3-B22 | GAGCCACTCCACGCACAG | HLA-B-Rxn 27^c^, 43^c^, 44 |
| 3-B23 | CTCGGTCAGTCTGTGCCTT | HLA-B-Rxn 29 |
| 3-B24 | TCTCGGTAAGTCTGTGCCTT | HLA-B-Rxn 29 |
| 3-B25 | CCTTGCCGTCGTAGGCGA | HLA-B-Rxn 31 |
| 3-B26 | TCGTAGGCGTCCTGGTGG | HLA-B-Rxn 32 |
| 3-B27 | GCCGCGGTCCAGGAGCT | HLA-B-Rxn 37^c^ |
| 3-B28 | GCAGGTTCCGCAGGCTCT | HLA-B-Rxn 38, 39 |
| 3-B29 | CAGGTATCTGCGGAGCCC | HLA-B-Rxn 45 |
| 3-B30 | CCTCCAGGTAGGCTCTCCA | HLA-B-Rxn 46 |
| 3-B32 | GGAGGAAGCGCCCGTCG | HLA-B-Rxn 48 |
| 3-B33 | CGTTCAGGGCGATGTAATCT | HLA-B-Rxn 49^c^, 50, 51^c^, 52 |
| 3-B34 | GTTGTAGTAGCGGAGCGCGA | HLA-B-Rxn 53 |
| 3-B35 | TTGTAGTAGCGGAGCAGGG | HLA-B-Rxn 54 |
| 3-B36 | TGTAGTAGCGGAGCGCGG | HLA-B-Rxn 54 |
| 3-B37 | TTGTAGTAGCCGCGCAGGT | HLA-B-Rxn 55 |
| C5'01-C368 | CACAGACTGACCGAGTGAG | HLA-Cw-Rxn 1^f^, 3^f^, 16^f^, 18^f^, 22^f^ |
| C5'03-C366 | CCGAGTGAACCTGCGGAAA | HLA-Cw-Rxn 2^f^, 4^f^, 5^f^, 20^f^, 21^f^, 23^f^, 24^f^ |
| C5'08-C367 | TACTACAACCAGAGCGAGGA | HLA-Cw-Rxn 6^f^, 9^f^, 10^f^, 11^f^ |
| C5'10-C130 | CCGCGGGTATGACCAGTC | HLA-Cw-Rxn 7^f^ |
| C5'12-C313 | GGACCGGGAGACACAGAAC | HLA-Cw-Rxn 8^g^ |
| C5'16-C165 | ACGACACGCAGTTCGTGCA | HLA-Cw-Rxn 12^g^, 13^g^ |
| C5'19-C159 | TACAACCAGAGCGAGGCCA | HLA-Cw-Rxn 14^f^ |
| C5'20-C160 | ACAACCAGAGCGAGGCCG | HLA-Cw-Rxn 15^f^ |
| C5'22-C369 | AGTCCAAGAGGGGAGCCG | HLA-Cw-Rxn 17^f^, 25^f^ |
| C5'24-C371 | CCACTCCATGAGGTATTTCTC | HLA-Cw-Rxn 19^f^ |
| C3'02-C315 | CCCCAGGTCGCAGCCAC | HLA-Cw-Rxn 1^h^ |
| C3'04-C145 | GAGCCACTCCACGCACTC | HLA-Cw-Rxn 2^f^ |
| C3'05-C389 | AGCGTCTCCTTCCCATTCTT | HLA-Cw-Rxn 3^f^, 14^f^, 15^f^ |
| C3'06-C143 | GCCCCAGGTCGCAGCCAA | HLA-Cw-Rxn 4^f^, 25^f^ |
| C3'07-C379 | CGCGCGCTGCAGCGTCTT | HLA-Cw-Rxn 5^f^, 11^f^ |
| C3'09-C127 | GGTCGCAGCCATACATCCA | HLA-Cw-Rxn 6^f^ |
| C3'11-C378 | CAGCCCCTCGTGCTGCAT | HLA-Cw-Rxn 7^f^ |
| C3'13-C184 | CGCACGGGCCGCCTCCA | HLA-Cw-Rxn 8^g^ |
| C3'14-C183 | CCCCAGGTCGCAGCCAG | HLA-Cw-Rxn 9^f^ |
| C3'15-C238 | GAGCCACTCCACGCACAG | HLA-Cw-Rxn 10^f^ |
| C3'17-C166 | GCGCAGGTTCCGCAGGC | HLA-Cw-Rxn 12^g^ |
| C3'18-C317 | TCTCAGCTGCTCCGCCGT | HLA-Cw-Rxn 13^g^ |
| C3'21-C126 | TGAGCCGCCGTGTCCGCA | HLA-Cw-Rxn 16^f^, 17^f^ |
| C3'23-C157 | CCGCCGTGTCCGCGGCA | HLA-Cw-Rxn 18^f^ |
| C3'25-C388 | GGTCGCAGCCAAACATCCA | HLA-Cw-Rxn 19^f^ |
| C3'26-C223 | GCCATACATCCTCTGGATGA | HLA-Cw-Rxn 20^f^ |
| C3'27-C382 | CCTCCAGGTAGGCTCTCAG | HLA-Cw-Rxn 21^f^ |
| C3'28-C146 | CCCTCCAGGTAGGCTCTCT | HLA-Cw-Rxn 22^f^, 23^f^ |
| C3'29-C377 | CCTCCAGGTAGGCTCTCCA | HLA-Cw-Rxn 24^f^ |
| DRB1-5'01 | TTGTGGCAGCTTAAGTTTGAAT | HLA-DRB1-Rxn 1, 2 |
| DRB1-5'02 | TCCTGTGGCAGCCTAAGAG | HLA-DRB1-Rxn 3^h^, 4^h^;  HLA-DRB1*15-Rxn 1^h^, 2^h^, 4^h^ |
| DRB1-5'03 | TACTTCCATAACCAGGAGGAGA | HLA-DRB1-Rxn 5^h^, 7^h^, 15^h^, 17^h^, 19^h^ |
| DRB1-5'04 | GTTTCTTGGAGCAGGTTAAACA | HLA-DRB1-Rxn 8^h^, 20^h^ |
| DRB1-5'05 | GTTTCTTGGAGTACTCTACGTC | HLA-DRB1-Rxn 13^h^, 16^h^, 18^h^ |
| DRB1-5'06 | GACGGAGCGGGTGCGGTA | HLA-DRB1-Rxn 6^h^, 11^h^ |
| DRB1-5'07 | GGTGCAGTTCCTGGAAAGACT | HLA-DRB1-Rxn 9^h^ |
| DRB1-5'08 | AGTACTCTACGGGTGAGTGTT | HLA-DRB1-Rxn 10^h^, 14^h^, 18^h^ |
| DRB1-5'10 | CGGTTGCTGGAAAGACGCG | HLA-DRB1-Rxn 12^h^ |
| DRB1-5'15 | GTGCGGTTCCTGGACAGAC | HLA-DRB1*15-Rxn 3^h^ |
| DRB1-5'51 | GTTTCTTGCAGCAGGATAAGTA | HLA-DRB1-Rxn 23^h^ |
| DRB1-5'52.1 | TTTCTTGGAGCTGCGTAAGTC | HLA-DRB1-Rxn 21^h^ |
| DRB1-5'52.2 | GTTTCTTGGAGCTGCTTAAGTC | HLA-DRB1-Rxn 21^h^ |
| DRB1-5'53 | GAGCGAGTGTGGAACCTGA | HLA-DRB1-Rxn 22^h^ |
| DRB1-3'01 | CCGCGCCTGCTCCAGGAT | HLA-DRB1-Rxn 3^h^, 23^h^;  HLA-DRB1*15-Rxn 3^h^ |
| DRB1-3'02 | AGGTGTCCACCGCGGCG | HLA-DRB1-Rxn 4^h^ |
| DRB1-3'03 | TGCAGTAGTTGTCCACCCG | HLA-DRB1-Rxn 5^h^ |
| DRB1-3'045 | TGTTCCAGTACTCGGCGCT | HLA-DRB1-Rxn 10^h^, 16^h^;  HLA-DQB1-Rxn 2^h^ |
| DRB1-3'047 | CTGCACTGTGAAGCTCTCAC | HLA-DRB1-Rxn 7^h^, 8^h^, 12^h^;  HLA-DRB1*15-Rxn 1^h^ |
| DRB1-3'048 | CTGCACTGTGAAGCTCTCCA | HLA-DRB1-Rxn 6^h^, 8^h^, 22^h^;  HLA-DRB1*15-Rxn 2^h^ |
| DRB1-3'06 | CTGGCTGTTCCAGTACTCCT | HLA-DRB1-Rxn 13^h^ |
| DRB1-3'079 | CCCGTAGTTGTGTCTGCACAC | HLA-DRB1-Rxn 9^h^, 11^h^ |
| DRB1-3'08 | CACTGTGAAGCTCTCCACAG | HLA-DRB1-Rxn 14^h^ |
| DRB1-3'10 | CCCGCTCGTCTTCCAGGAT | HLA-DRB1-Rxn 2^h^, 15^h^ |
| DRB1-3'11 | TCTGCAATAGGTGTCCACCT | HLA-DRB1-Rxn 18^h^ |
| DRB1-3'12 | TCCACCGCGGCCCGCC | HLA-DRB1-Rxn 19^h^ |
| DRB1-3'13 | CTGTTCCAGGACTCGGCGA | HLA-DRB1-Rxn 21^h^ |
| DRB1-3'14 | GCTGTTCCAGTACTCGGCAT | HLA-DRB1-Rxn 10^h^, 21^h^;  HLA-DQB1-Rxn 5^h^, 6^h^ |
| DRB1-3'15 | CCGCCTCTGCTCCAGGAG | HLA-DRB1-Rxn 1^h^ |
| DRB1-3'1506 | CCGCCCCAGCTCCGTCG | HLA-DRB1*15-Rxn 4^h^ |
| DRB1-3'16 | CCGCGGCGCGCCTGTCT | HLA-DRB1-Rxn 23^h^ |
| DRB1-3'17 | CCCGCCTGTCTTCCAGGAA | HLA-DRB1-Rxn 17^h^ |
| DRB1-3'19 | CTGTTCCAGTGCTCCGCAG | HLA-DRB1-Rxn 20^h^ |
| DQB-5'01 | CGGAGCGCGTGCGGGG | HLA-DQB1-Rxn 1^h^ |
| DQB-5'02 | TGCGGGGTGTGACCAGAC | HLA-DQB1-Rxn 2^h^, 3^h^ |
| DQB-5'03 | GCCATGTGCTACTTCACCAAT | HLA-DQB1-Rxn 4^h^ |
| DQB-5'04 | CGTGCGTCTTGTGACCAGAT | HLA-DQB1-Rxn 5^h^ |
| DQB-5'05 | GGAGCGCGTGCGTCTTGTA | HLA-DQB1-Rxn 6^h^ |
| DQB-5'06 | CGTGTACCAGTTTAAGGGCA | HLA-DQB1-Rxn 7^h^ |
| DQB-5'07 | GTGCGTCTTGTGAGCAGAAG | HLA-DQB1-Rxn 8^h^ |
| DQB-5'08 | GACGGAGCGCGTGCGTCT | HLA-DQB1-Rxn 9^h^, 11^h^, 12^h^ |
| DQB-5'09 | GACGGAGCGCGTGCGTTA | HLA-DQB1-Rxn 10^h^ |
| DQB-5'10 | CACCAACGGGACCGAGCT | HLA-DQB1-Rxn 13^h^ |
| DQB-5'11 | CACCAACGGGACCGAGCG | HLA-DQB1-Rxn 14^h^ |
| DQB-3'01 | GCTGTTCCAGTACTCGGCAA | HLA-DQB1-Rxn 1^h^ |
| DQB-3'03 | GCGGCGTCACCGCCCGA | HLA-DQB1-Rxn 3^h^ |
| DQB-3'04 | CACCGTGTCCAACTCCGCT | HLA-DQB1-Rxn 4^h^ |
| DQB-3'06 | GCAGGATCCCGCGGTACC | HLA-DQB1-Rxn 7^h^ |
| DQB-3'07 | GCAAGGTCGTGCGGAGCT | HLA-DQB1-Rxn 8^h^ |
| DQB-3'08 | CTGTTCCAGTACTCGGCGG | HLA-DQB1-Rxn 9^h^ |
| DQB-3'09 | AGTACTCGGCGTCAGGCG | HLA-DQB1-Rxn 10^h^, 11^f^ |
| DQB-3'10 | CTGTTCCAGTACTCGGCGT | HLA-DQB1-Rxn 12^h^ |
| DQB-3'11 | GGTAGTTGTGTCTGCATACG | HLA-DQB1-Rxn 13^h^, 14^h^ |

^a^Primer concentration at 2.5μM unless otherwise indicated

^b^Primer concentration 400nM

^c^Primer concentration 5μM

^d^Primer concentration 7.5μM

^e^Primer concentration 10μM

^f^Primer concentration 6μM

^g^Primer concentration 4μM

^h^Primer concentration 2μM
