# Supplementary material for: A second major histocompatibility complex susceptibility locus for multiple sclerosis
Source: Ann Neurol. 2007 Mar;61(3):228–36. doi: 10.1002/ana.21063 (PMC2737610; doi:10.1002/ana.21063)
Supplement: Supplementary file 3 [file ana0061-0228-SD3.doc]

**Table S3**. This series of tables indicates the allele counts and frequencies for the classical HLA loci in the screening and extension phases.

| HLA-A allele counts and frequencies in the screening phase. | | |
| --- | --- | --- |
| Allele (groups) | Transmitted / N (%) | Non-Transmitted / N (%) |
| 01 | 164 (18.4) | 170 (19.1) |
| 02 | 223 (25.1) | 277 (31.1) |
| 03 | 172 (19.3) | 107 (12.0) |
| 11 | 62 (7.0) | 56 (6.3) |
| 23 | 19 (2.1) | 10 (1.1) |
| 24 | 79 (8.9) | 86 (9.7) |
| 25 | 19 (2.1) | 12 (1.3) |
| 26 | 13 (1.5) | 23 (2.6) |
| 29 | 30 (3.4) | 45 (5.1) |
| 30 | 25 (2.8) | 9 (1.0) |
| 31 | 23 (2.6) | 22 (2.5) |
| 32 | 29 (3.3) | 28 (3.1) |
| 68 | 21 (2.4) | 33 (3.7) |
| Other | 11 (1.1) | 12 (1.4) |

| HLA-Cw allele counts and frequencies in the screening phase. | | |
| --- | --- | --- |
| Allele (groups) | Transmitted / N (%) | Non-Transmitted / N (%) |
| 01 | 30 (3.4) | 33 (3.7) |
| 02 | 25 (2.8) | 38 (4.3) |
| 04 | 78 (8.7) | 51 (5.7) |
| 05 | 62 (7.0) | 104 (11.7) |
| 06 | 80 (9.0) | 73 (8.2) |
| 08 | 31 (3.5) | 32 (3.6) |
| 12 | 28 (3.1) | 39 (4.4) |
| 15 | 27 (3.0) | 19 (2.1) |
| 16 | 30 (3.4) | 46 (5.2) |
| 0302/0304/0305/0306 | 50 (5.6) | 84 (9.4) |
| 0303/0311/0312 | 41 (4.6) | 49 (5.5) |
| 0701/0706 | 136 (15.2) | 145 (16.3) |
| 0702/0710 | 256 (28.7) | 150 (16.8) |
| 0704/0711/0712 | 5 (0.6) | 18 (2.0) |
| Other | 13 (1.4) | 11 (1.1) |

| HLA-B allele counts and frequencies in the screening phase. | | |
| --- | --- | --- |
| Allele (groups) | Transmitted / N (%) | Non-Transmitted / N (%) |
| 07 | 248 (27.9) | 138 (15.5) |
| 08 | 104 (11.7) | 122 (13.7) |
| 13 | 13 (1.5) | 13 (1.5) |
| 18 | 46 (5.2) | 30 (3.4) |
| 27 | 33 (3.7) | 36 (4.0) |
| 35 | 57 (6.4) | 43 (4.8) |
| 37 | 17 (1.9) | 8 (0.9) |
| 38 | 2 (0.2) | 18 (2.0) |
| 39 | 19 (2.1) | 11 (1.2) |
| 44 | 97 (10.9) | 161 (18.1) |
| 49 | 11 (1.2) | 5 (0.6) |
| 50 | 10 (1.1) | 11 (1.2) |
| 51 | 44 (4.9) | 54 (6.1) |
| 57 | 31 (3.5) | 32 (3.6) |
| 1402/1403 | 21 (2.4) | 22 (2.5) |
| 1501/1504/1505/1506 | 48 (5.4) | 63 (7.1) |
| 4001 | 43 (4.8) | 58 (6.5) |
| 4002/4003/4004/4006 | 4 (0.4) | 14 (1.6) |
| Other | 42 (4.8) | 51 (5.7) |

| HLA-DRB1 allele counts and frequencies in the screening phase. | | |
| --- | --- | --- |
| Allele (groups) | Transmitted / N (%) | Non-Transmitted / N (%) |
| 01 ^a^ | 115 (6.2) | 185 (9.9) |
| 0103 | 36 (1.9) | 29 (1.6) |
| 04 | 268 (14.4) | 336 (18.1) |
| 07 | 179 (9.6) | 253 (13.6) |
| 08 | 58 (3.1) | 54 (2.9) |
| 09 | 13 (0.7) | 22 (1.2) |
| 11 | 101 (5.4) | 152 (8.2) |
| 12 | 25 (1.3) | 23 (1.2) |
| 13 | 170 (9.1) | 192 (10.3) |
| 14 | 18 (1) | 47 (2.5) |
| 1501 | 616 (33.1) | 245 (13.2) |
| 1502 | 10 (0.6) | 17 (0.9) |
| 16 | 22 (1.2) | 26 (1.4) |
| 17 | 225 (12.1) | 269 (14.5) |
| Other | 4 (0.3) | 10 (0.5) |

^a^ This group includes all 01 alleles except 0103.

| HLA-DQB1 allele counts and frequencies in the screening phase. | | |
| --- | --- | --- |
| Allele (groups) | Transmitted / N (%) | Non-Transmitted / N (%) |
| 02 | 357 (19.7) | 445 (24.6) |
| 03 | 490 (27) | 604 (33.3) |
| 04 | 53 (2.9) | 52 (2.9) |
| 05 | 170 (9.4) | 288 (15.9) |
| 06 | 742 (41) | 423 (23.3) |

| HLA-A allele counts and frequencies in the extension phase. | | |
| --- | --- | --- |
| Allele (groups) | Cases / N (%) | Controls / N (%) |
| 01 | 420 (19.5) | 959 (18.4) |
| 02 | 519 (24.1) | 1584 (30.4) |
| 03 | 406 (18.8) | 783 (15.0) |
| 11 | 136 (6.3) | 310 (5.9) |
| 23/24 ^a^ | 229 (10.6) | 451 (8.6) |
| 25/26 ^a^ | 95 (4.4) | 228 (4.4) |
| 29/30/31/32/33 ^a^ | 280 (13.0) | 708 (13.5) |
| 68 | 70 (3.2) | 192 (3.7) |
| Other | 3 (0.1) | 3 (0.1) |

^a^ As these allele groups were not resolved in each of the contributing data sets they were down grouped as shown.

| HLA-Cw allele counts and frequencies in the extension phase. | | |
| --- | --- | --- |
| Allele (groups) | Cases / N (%) | Controls / N (%) |
| 01 | 61 (2.6) | 195 (3.7) |
| 02 | 77 (3.3) | 212 (4.1) |
| 04 | 217 (9.2) | 452 (8.7) |
| 05 | 169 (7.2) | 615 (11.8) |
| 06 | 205 (8.7) | 460 (8.8) |
| 07 ^b^ | 1008 (42.8) | 1791 (34.3) |
| 08 | 102 (4.3) | 203 (3.9) |
| 12 | 71 (3.0) | 166 (3.2) |
| 14 | 24 (1.0) | 36 (0.7) |
| 15 | 68 (2.9) | 95 (1.8) |
| 16 | 86 (3.7) | 248 (4.7) |
| 17 | 15 (0.6) | 24 (0.5) |
| 32/33 ^a^ | 252 (10.7) | 719 (13.8) |
| Other | 1 (0.0) | 2 (0.0) |

^a^ As these allele groups were not resolved in each of the contributing data sets they were down grouped as shown.

^b^ In the screening phase the 7 alleles were resolved into three subgroups.

| HLA-B allele counts and frequencies in the extension phase. | | |
| --- | --- | --- |
| Allele (groups) | Cases / N (%) | Controls / N (%) |
| 07 | 526 (24.4) | 1072 (14.6) |
| 08 | 288 (13.4) | 982 (13.4) |
| 13 | 41 (1.9) | 136 (1.9) |
| 14 | 84 (3.9) | 289 (3.9) |
| 15 | 131 (6.1) | 531 (7.3) |
| 18 | 106 (4.9) | 284 (3.9) |
| 27 | 77 (3.6) | 332 (4.5) |
| 35 | 143 (6.6) | 486 (6.6) |
| 37 | 51 (2.4) | 103 (1.4) |
| 38/39 ^a^ | 42 (1.9) | 179 (2.5) |
| 40 | 122 (5.7) | 502 (6.9) |
| 41 | 14 (0.6) | 37 (0.5) |
| 44/45 ^a^ | 255 (11.8) | 1342 (18.3) |
| 47 | 4 (0.2) | 23 (0.3) |
| 49/50 ^a^ | 48 (2.2) | 145 (2.0) |
| 51/52 ^a^ | 119 (5.5) | 342 (4.7) |
| 53 | 4 (0.2) | 18 (0.2) |
| 55/56 ^a^ | 27 (1.3) | 160 (2.2) |
| 57/58 ^a^ | 74 (3.4) | 354 (4.8) |
| Other | 0 (0.0) | 3 (0.1) |

^a^ As these allele groups were not resolved in each of the contributing data sets they were down grouped as shown.

| HLA-DRB1 allele counts and frequencies in the extension phase. | | |
| --- | --- | --- |
| Allele (groups) | Cases / N (%) | Controls / N (%) |
| 01 ^a^ | 136 (5.7) | 758 (10.4) |
| 0103 | 62 (2.6) | 123 (1.7) |
| 04 | 339 (14.1) | 1447 (19.8) |
| 07 | 253 (10.5) | 1065 (14.5) |
| 08 | 69 (2.9) | 143 (2.0) |
| 09 | 17 (0.7) | 101 (1.4) |
| 10 | 10 (0.4) | 42 (0.6) |
| 11/12 ^b^ | 136 (5.7) | 567 (7.7) |
| 13/14 ^b^ | 191 (8.0) | 860 (11.7) |
| 15 | 832 (34.6) | 1107 (15.1) |
| 16 | 18 (0.7) | 52 (0.7) |
| 17 | 339 (14.1) | 1055 (14.4) |

^a^ This allele group includes all 01 alleles except 0103.

^b^ As these allele groups were not resolved in each of the contributing data sets they were down grouped as shown.

| HLA-DQB1 allele counts and frequencies in the extension phase. | | |
| --- | --- | --- |
| Allele (groups) | Cases / N (%) | Controls / N (%) |
| 02 | 500 (22.7) | 1825 (24.9) |
| 03 | 557 (25.2) | 2513 (34.3) |
| 04 | 58 (2.6) | 135 (1.9) |
| 05/06 ^a^ | 1091 (49.5) | 2845 (38.9) |

^a^ As these allele groups were not resolved in each of the contributing data sets they were down grouped as shown.
